# Influence of Various Nanomaterials on the Rheology and Hydration Kinetics of Oil Well Cement

**DOI:** 10.3390/ma16196514

**Published:** 2023-09-30

**Authors:** Michael Boniface Baragwiha, Kenedy Geofrey Fikeni, Yukun Zhao, Guodong Cheng, Han Ge, Xueyu Pang

**Affiliations:** 1 School of Petroleum Engineering, China University of Petroleum (East China), Qingdao 266580, China; 2 National Key Laboratory of Deep Oil and Gas, China University of Petroleum (East China), Qingdao 266580, China

**Keywords:** nanomaterials, polycarboxylate superplasticizer, diutan gum, low-density slurry, rheological properties, hydration kinetics

## Abstract

Nanomaterials have great potential to influence the properties of cement-based materials due to their small particle size and large specific surface area. The influences of Nano-SiO_2_ (NS), gamma-nano-Al_2_O_3_ (GNA), alpha-nano-Al_2_O_3_ (ANA), and nano-TiO_2_ (NT) on the rheology and hydration kinetics of class G cement at 30 °C were investigated in this study. The nanomaterials were added in dry powder form at dosages of 1, 2, 3, 5, and 7% by weight of cement (bwoc), and their dispersion was accomplished using polycarboxylate superplasticizer (PCE) at a dosage of 1.6% bwoc. PCE provides a uniform dispersion of nanoparticles in the cement matrix, enhancing the efficiency of nanomaterials. The w/c ratio varied between 0.718 and 0.78 to form a constant-density slurry of 1.65 g/cm^3^. Our test results showed that NS and GNA caused significant increases in the rheology of the cement slurry, with this effect increasing with dosage, while ANA and NT tended to reduce the rheology of the slurry. Compared to a well-suspended and well-dispersed cement slurry generated by the use of PCE and diutan gum, all nanomaterials can accelerate early hydration by reducing the induction time, with GNA having the strongest influence, while NS was the only nanomaterial that further increased the long-term hydration heat release at 7 days. The stronger effect of NS and GNA on the cement slurry properties can be attributed to their higher chemical reactivity. The dosage effect on total hydration extent was relatively strong for ANA, NT, and NS from 3% to 5% but weak for GNA in the range from 3% to 7%.

## 1. Introduction

Cementing is one of the key tasks in well construction, the main objective of which is to provide zonal isolation along the well and mechanical support to the wellhead equipment throughout the life of the wellbore [1,2]. To achieve the required well integrity, various admixtures can be added to tailor the cement properties, both fresh (short term) and hardened (long term) [3]. The properties of a fresh cement slurry (such as rheology, density, stability, etc.) play a major role in cement slurry placement in the annulus. The quality of cement slurry placement determines the mechanical and durability properties of a hardened cement paste [4,5].

Recently, nanotechnology research has attracted interest in the oil and gas industry due to the unique properties of nanomaterials. Nanomaterials are materials that have nanoparticles (NPs) embedded in their structures. NPs are ultrafine particles with sizes ranging from 1 to 100 nm in two or three dimensions, resulting in a very large specific surface area and, as a result, high chemical reactivity [6,7,8]. Nanomaterials are produced using two approaches: top-down and bottom-up approaches. The top-down approach involves size reduction, where larger materials are broken into smaller sizes using methods such as mechanical attrition and etching techniques. Meanwhile, in the bottom-up approach, smaller materials (atoms or molecular components) are assembled to form larger materials through techniques such as synthesis and chemical formulation [5,9].

In the oil and gas industry, nanomaterials find applications in drilling, cementing, enhanced oil recovery, etc. In well cementing, nanomaterials have been reported to be able to influence the properties of fresh cement slurry, hydration kinetics, and the properties of hardened cement paste [6]. Nanomaterials influence the properties of fresh cement slurry, such as pumpability, workability, consistency, compatibility, stability, etc. [8]. In terms of hydration kinetics, they have been reported to accelerate the rate of cement hydration by providing additional nucleation sites for calcium silicate hydrate (C-S-H) gel precipitation and growth [10]. The accelerated hydration kinetics reduce thickening time, allowing for early strength development [11]. The addition of nanomaterials modifies the microstructure of the hydration products, consequently improving the mechanical and durability properties of the hardened cement paste. Because of their ultrafine sizes, they act as nanofilling agents that seal the micro- and nanopores in C-S-H gel, thus densifying and refining the micro- and nanopores (reducing the porosity and permeability), resulting in a more compact pore structure with enhanced strength and durability [9,12].

Since nanomaterials accelerate the rate of cement hydration, they can be used in cementing low-temperature wellbore columns encountered when drilling offshore and in shallow formations. Low temperatures can cause long wait-on-cement times due to the extended setting time and late strength build-up due to the delay in cement hydration kinetics, which can compromise well integrity. A long wait-on-cement time causes an extended drilling time and, as a result, increased operational cost [13,14]. Mostly, inorganic salts such as calcium chloride (CaCl_2_) have been used as conventional accelerators; however, these can increase the permeability of the set cement, potentially affecting the robustness of the hardened cement [15]. The chloride accelerators may also corrode the casing [15]. These problems associated with conventional accelerators are among the factors driving the search for alternative additives for well cementing.

Nanomaterials are generally categorized as carbon-based (e.g., carbon nanotube, graphene, etc.), inorganic-based (e.g., metal oxides), or organic-based (e.g., polymeric NPs, dendrimers, etc.) nanomaterials [5]. The most commonly researched nanomaterials in well cementing applications include nano-SiO_2_, nano-Al_2_O_3_, nano-Fe_2_O_3_, nano-TiO_2_, nano-CaCO_3_, and nano-clays [6,16]. Nano-SiO_2_ is the most researched among the oxides, and it has been reported to have a much greater influence on oil well cement properties than the others. When added to cement slurries, nano-SiO_2_ speeds up early-stage cement hydration without appreciably slowing it down at later ages [15]. The effectiveness of silica particles as accelerators is influenced by their size and surface area, as well as their pozzolanic reactivity [14]. Nano-SiO_2_ can accelerate cement hydration through a combination of three mechanisms: (1) its large surface area provides nucleation sites for C-S-H gel, thereby accelerating C-S-H precipitation and growth [17]; (2) it reacts (pozzolanic reaction) with Portlandite (CH) crystals to form C-S-H gel (referred to as secondary C-S-H gel) [16]; and (3) the particles act as early C-S-H seeds [14,18]. 

The addition of nano-Al_2_O_3_ to cement slurry has been reported to accelerate early cement hydration kinetics, resulting in improved mechanical properties in the hardened cement [19]. The accelerating effect of nano-alumina on cement hydration can be viewed from two perspectives: the physical (filler) effect and the chemical effect. From a physical perspective, the presence of nano-alumina provides a greater surface area for the precipitation and growth of C-S-H gel, accelerating C_3_S dissolution. From a chemical perspective, the presence of nano-alumina accelerates the aluminate (C_3_A) reaction, as evidenced by an increased shoulder peak in the heat flow curve. It has been reported that nano-alumina (particularly the gamma phase) has a high dissolution rate, which contributes to the formation of additional ettringite at an early age, thus promoting the consumption of gypsum and thereby accelerating the C_3_A reaction [20]. It has also been reported that the presence of nano-alumina promotes the reaction between alumina and calcium hydroxide (CH), improving the strength of cement-based materials at later ages [5,21,22]. The addition of nano-TiO_2_ to cement slurry was reported to have similar effect in accelerating cement hydration and to improve compressive strength by the authors of [23].

The use of nanomaterials is associated with two challenges, particularly at high dosages. The first challenge is attributed to the agglomeration effects caused by poor dispersion. Because of their strong inter-particle attraction, nanomaterials tend to agglomerate (form clusters) as the dosage increases, negatively affecting the workability of cement slurry, hydration, and compressive strength development [8,24]. The problem is more severe when dealing with cement slurries with low water/cement ratios [25]. Another challenge is the reduction in slurry fluidity. Nanomaterials have large surface areas; therefore, they will adsorb large quantities of water, negatively affecting the workability of fresh cement slurry [6]. These two challenges will negatively affect the mechanical properties and durability of hardened cement. Various studies have reported decreases in the mechanical properties of cement-based materials at high dosages of nanomaterials: Heikal et al. [26] observed decreases in compressive strength when nano-Al_2_O_3_ content exceeded 2–6% (bwoc); Ma et al. [27] found decreases in flexural and tensile strengths when the dosage of nano-TiO_2_ was greater than 3% (bwoc); Maagi et al. [24] reported decreases in compressive strength when nano-SiO_2_ dosage was above 3% (bwoc). Significant efforts have been made to find the optimal dosages of nanomaterials. Despite extensive research, there are no explicit conclusions regarding the optimal dosages of nanomaterials, since they are a function of particle size as well as material composition. Therefore, the focus of this study was to evaluate the influence of material composition and dosage on the rheological and hydration properties of oil well cement using four types of nanomaterials (nano-SiO_2_, gamma-nano-Al_2_O_3_, alpha-nano-Al_2_O_3_, and nano-TiO_2_). 

## 2. Materials and Methods

### 2.1. Materials

#### 2.1.1. Cement and Other Additives

**Figure 1:**
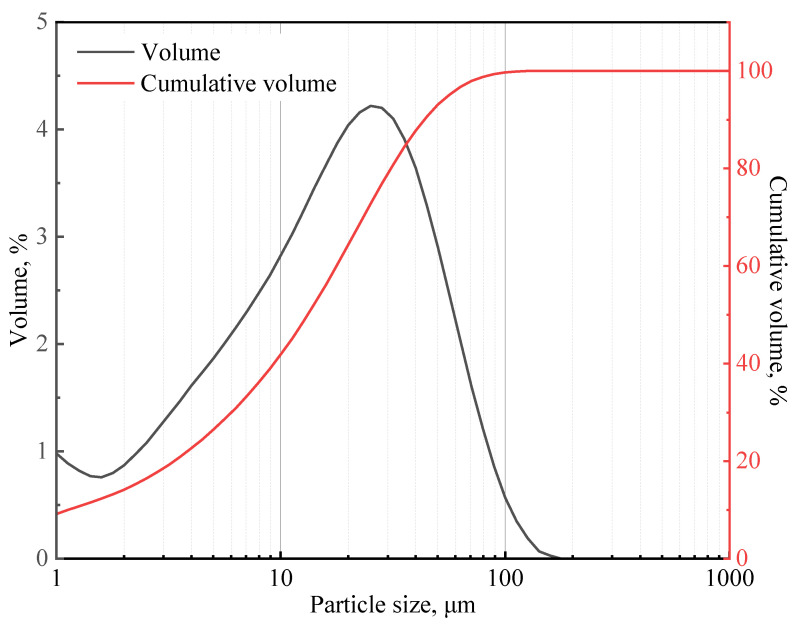
Class G cement particle size distribution curve.

In this study, class G cement from Shengwei Special Cement Co., Ltd. was used. This cement has a specific gravity of 3.17. Table 1 shows the chemical composition of the class G cement, which was determined using X-ray diffraction (XRD) Rietveld method. The particle size distribution of the cement (presented in Figure 1) was measured using a particle size analyzer (Mastersizer 2000), and the median particle size is 14.77 μm.

Antifoam agent G603, provided by CNPC Tianjin Bo-Xing Engineering Science & Technology Company Ltd., (with a specific gravity of 0.96) was used to prevent the formation of air bubbles in the cement slurry. G603 was used in all slurry designs at a constant dosage of 0.2% bwoc. Diutan gum (DG), obtained from CP Kelco Biological Co., Ltd., was used as a suspension agent to improve slurry sedimentation stability. DG has a specific gravity of 1.47, and it was used at dosages ranging from 0 to 0.02% bwoc during this study. Because the use of nanomaterials (particularly at high dosages) may cause particle agglomeration, a polycarboxylate superplasticizer (PCE) was used to ensure the proper dispersion of the nanomaterials and cement particles during mixing. The PCE was supplied by China Construction Western Building Materials Science Research Institute Co., Ltd., and it had an 18% solid content and a measured density of 0.986 g/cm^3^. The dispersion effect exerted by the PCE also improved the slurry workability. 

#### 2.1.2. Nanomaterials 

**Figure 2:**
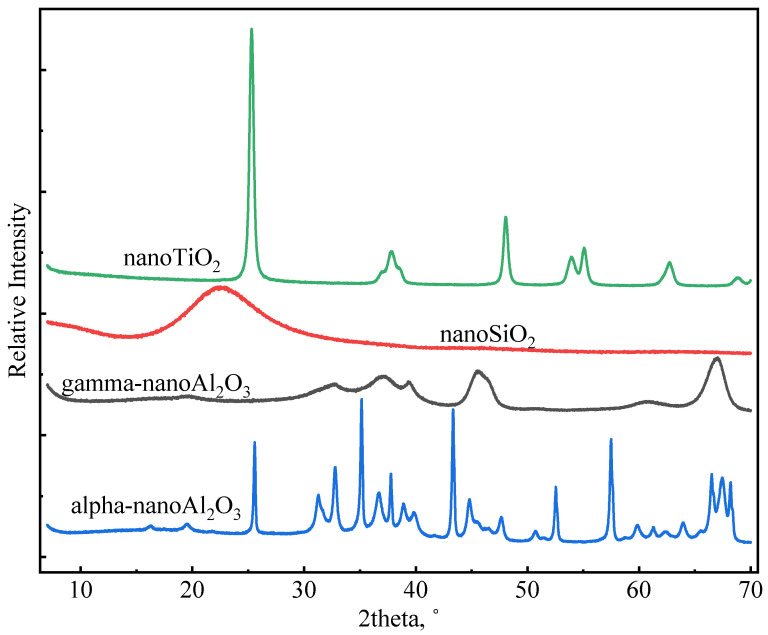
XRD profiles of nanomaterials.

Nano-SiO_2_ (NS), gamma-nano-Al_2_O_3_ (GNA), alpha-nano-Al_2_O_3_ (ANA), and nano-TiO_2_ (NT) were purchased from Shanghai Macklin Biochemical Technology Co., Ltd. All four nanomaterials were provided in solid state. The densities of the nanomaterials were determined using an automatic density analyzer (ULTRAPYC 1200e). Table 2 presents the measured density (g/cm^3^) of the nanomaterials used in this study. Table 2 also shows the particle size (nm) and surface area (m^2^/g) of the nanomaterials (as provided by the manufacturer). The higher surface area of GNA compared to the other materials may be associated with its high-porosity structure. Figure 2 shows XRD profiles of nanomaterials measured using the Malvern PANalytical X-ray Diffractometer (Model AerisX). Clearly, NS and GNA are mostly amorphous, whereas ANA and NT exhibit strong crystallinity. In general, amorphous materials are more reactive than crystalline materials due to the presence of structural defects. Chen [28] showed that the addition of 5% and 10% bwoc of nano-TiO_2_ resulted in a significant increase in cement’s heat of hydration. However, whether the use of high dosages of nanomaterials in general has a better impact on cement properties has not been comprehensively explored. Therefore, this study made use of five dosages of nanomaterials in various slurry designs: 1%, 2%, 3%, 5%, and 7% bwoc. 

#### 2.1.3. Slurry Design

The addition of nanomaterials to cement requires a high amount of water to maintain the flowability of the slurry [29]. Maagi et al. [29] found that cement slurries containing nano-SiO_2_ at a dosage of 4% bwoc and a water/cement (w/c) ratio of 0.45 thickened instantly before any tests could be performed. As a result, nanomaterials are better suited to slurries with high w/c ratios. However, the use of a very high w/c ratio is generally associated with severe particle settlement [30]. Nanomaterials are among the additives that can be used to mitigate the settlement/sedimentation problem of the so-called “water-extended slurries”. Without the use of various low-density materials, the minimum density that can be achieved for water-extended slurries is approximately 1.6 g/cm^3^ [31]. During this study, all of the slurries were designed to have the same density of 1.65 g/cm^3^. Constant-density slurries were prepared by slightly varying w/c ratios depending on the density and dosages of particular additives. 

Table 3 shows the detailed slurry designs employed in this study. The masses of various additives are expressed as percentages by weight of cement (%bwoc). Three control slurries—control-1 (C-1), control-2 (C-2), and control-3 (C-3)—were designed to evaluate the influences of PCE and DG on slurry performance; they all had similar designs, although control-2 contained no PCE, and control-3 contained no DG. A constant defoamer (G603) dosage of 0.2% and PCE dosage of 1.6% were used throughout these tests, except for control-2 (which contained no PCE). The dosage of DG varied between different slurries depending on the need for particle suspension to obtain relatively stable slurries. The DG dosage was 0.02% in control slurries control-1 and control-2, but it was reduced to 0.01% in some of the nanomaterial-enriched slurries (all ANA-enriched and NT-enriched slurries, as well as the low-dosage NS-enriched and GNA-enriched slurries). No DG was needed in the high-dosage (3–7%) NS-enriched and GNA-enriched slurries due to the superior suspension ability of the nanomaterials.

### 2.2. Methods

#### 2.2.1. Mixing

**Figure 3:**
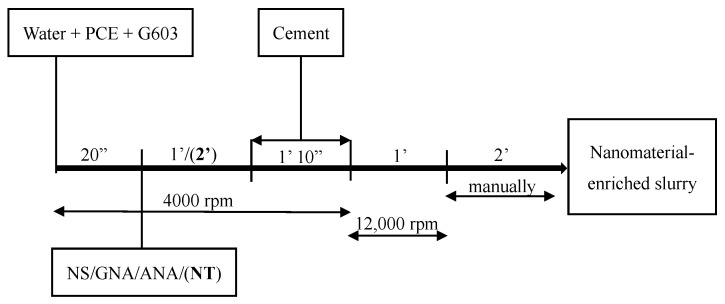
Cement slurry mixing procedure.

In order to maximize the performance of PCE, a mixing procedure similar to that described by the authors of [32] was applied in this study. As described in Figure 3, G603 and PCE were added to the mixing water and mixed together at 4000 rev/min for 20 s, and the nanomaterials were subsequently dispersed for 1 min (or 2 min for NT) at 4000 rev/min. Then, cement was continuously added for 1 min and 10 s at the same speed of 4000 rev/min before blending them together at 12,000 rev/min for 1 min. At last, the mixture was mixed manually for 2 min by using a spatula. Slurry C-2 (without PCE) was mixed according to API mixing procedures [33].

#### 2.2.2. Stability Test

According to API standards [33], slurry stability evaluations involve two types of tests: free fluid test and sedimentation test. Ideally, a well cementing slurry should have zero free fluid and uniform density for field applications. As discussed earlier, nanomaterials are generally believed to enhance slurry stability. During this study, different dosages of nano-SiO_2_ were added to control-3 to study the effect of nanomaterials on the stability of oil well cement slurries. The free fluid test was conducted by pouring a cement slurry into a 250 mL graduated cylinder and measuring the amount (%) of free-standing fluid on top of the cement slurry after 2 h of curing in a water bath at 30 °C. The sedimentation stability test was conducted by pouring a cement slurry into a stainless-steel mold with an internal diameter of 25 mm and a height of 150 mm. After setting in a water bath at 30 °C, the cement was demolded and cut into 6 sections with equal height for density measurement.

#### 2.2.3. Rheology Test

Rheology tests are used to describe the workability, pumpability, consistency, flowability, and stability of a cement slurry [34,35], aiding proper cement placement [35]. It is crucial to characterize and optimize the rheological properties of cement slurries in order to improve mud clean-up and provide good zonal isolation [35]. The rheology test conducted for this study was performed at atmospheric pressure using an automatic rotational viscometer (model NXNJ) from Tianjin NITHONS Technology CO., Ltd., Tianjin, China. Approximately 360 mL of slurry was added up to the scribed line of the viscometer cup. Prior to the rheology test, the slurry was conditioned for 30 min at 30 °C and atmospheric pressure using an atmospheric pressure consistometer. By using a water bath, the viscometer cup was warmed to 30 °C prior to its use in order to maintain slurry temperature during the test. Shear rate was increased from 3 rpm to 300 rpm and then decreased back to 3 rpm according to standard API test procedures [34]. The average dial reading during the ramp-up and ramp-down periods was employed to calculate the shear stress. Moreover, gel strength was measured immediately after determining the rheological properties. Firstly, the slurry was reconditioned in the viscometer for 1 min at 300 rpm to disperse the gels in order to facilitate a better gel strength measurement. Approximately, 10 s after the rotor stopped, rotation started at 3 rpm, and the maximum dial recording multiplied by a factor applicable for the specific rotor, bob, and spring configuration was obtained as the 10 s gel strength. Similarly, 10 min after the rotor stopped (from measuring 10 s gel strength), rotation started at 3 rpm, and the maximum dial recording multiplied by the same applicable factor was obtained as the 10 min gel strength.

Various rheological models are used to characterize fluid flow behaviors, such as the Bingham plastic model, power law model, Herschel–Bulkley model, Robertson stiff model, Casson model, Carreau model, Ellis model, Cross model, Vocadlo model, and Vipulanandan model [33,36,37]. The Bingham plastic model and power law model are the most commonly used rheological models for cement slurries, and they are shown via Equations (1) and (2), respectively [38]:

(1) $$\tau =\mu_{p}\gamma +\tau_{y}$$

(2) $$\tau =K\gamma^{n}$$

where τ is shear stress, γ is shear rate, $\mu_{p}$ is the plastic viscosity, $\tau_{y}$ is yield stress, K is the consistency index, and “n” is the power law behavior index.

#### 2.2.4. Hydration Test

An Isothermal Calorimeter (I-CAL FLEX) was used to evaluate the influence of nanomaterials on cement hydration kinetics. After mixing, approximately 5.12 g to 5.29 g of a cement slurry was added to a glass vial, which was then sealed and inserted into the calorimeter. The tests were carried out for 7 days at 30 °C. It should be noted that the first hour of data recording was ignored because it takes approximately one hour for the samples to reach an equilibrium state (test temperature). Hydration heat tests were conducted for all control slurries and slurries with high dosages (3–7%) of nanomaterials.

## 3. Results and Discussion

### 3.1. Stability Test

The slurry with no suspension aid additives, i.e., DG and nanomaterials (control-3) had the highest amount of free fluid of 1.6%. Sedimentation analysis was performed on this slurry (control-3) and the NS-enriched slurries with dosages of 2%, 5%, and 7%, which were designated as C-3, NS-2, NS-5, and NS-7, respectively. The height of the C-3, NS-2, NS-5, and NS-7 specimens after setting were 101.73 mm, 138.76 mm, 148.29 mm, and 149.77 mm, respectively. The results suggest that additional significant sedimentation may occur in the unstable slurry (control-3) after the free fluid test period of 2 h. Clearly, at high NS dosages, the specimen height approaches the mold height (150 mm), indicating a reduced settling effect. Density difference (Δρ) was used to further investigate the sedimentation of cement specimens [33], whereby the density difference (Δρ) was normalized by the average density (ρ_av_) of the set cement rather than the slurry density. This is because the density of cement slurry increases naturally upon setting due to chemical shrinkage during cement hydration [15]. Table 4 shows sedimentation test results for slurries control-3, NS-2, NS-5, and NS-7, with w/ratios c of 0.718, 0.72, 0.742, and 0.752, respectively, which were varied to obtain a constant slurry density of 1.65 g/cm^3^. Based on the results, slurry control-3 showed severe settling, with a maximum density difference of 56.89%. Upon the addition of NS by 2% dosage in NS-2, the slurry stability was improved to a great extent. However, NS-2 showed a clear increase in density from the top to the bottom segment, with a maximum density difference of 4.75%, and large deviations were observed for the top segment and the bottom segment. When NS dosage was increased to 5% in NS-5, the specimen density became more uniform, with a maximum density difference of 1.8%. When NS dosage was further increased to 7% in NS-7, the uniformity of specimen density was further improved, with a maximum density difference of 0.7%. Clearly, adding and increasing the dosage of NS can significantly improve the suspension stability of cement slurries.

### 3.2. Rheology

**Figure 4:**
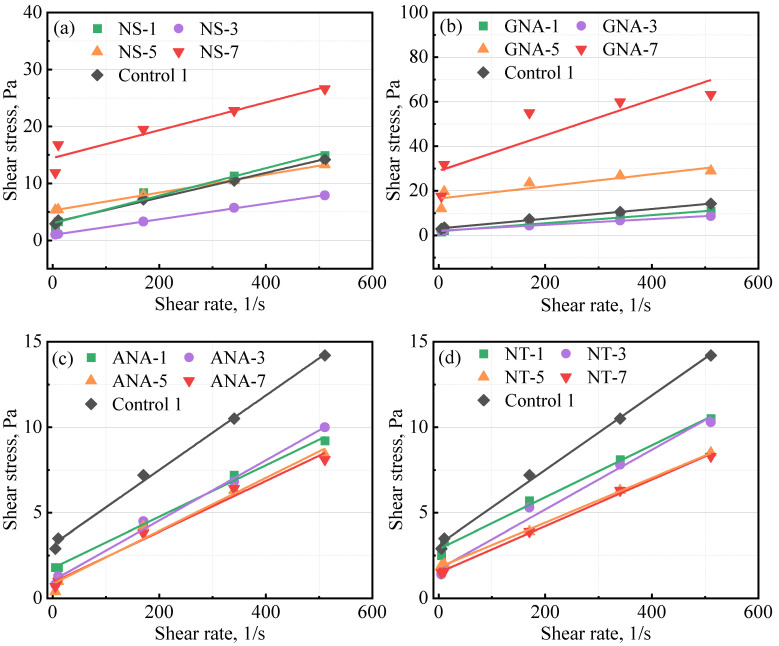
Influence of nanomaterials on class G cement rheology: (**a**) NS-enriched slurries; (**b**) GNA-enriched slurries; (**c**) ANA-enriched slurries; (**d**) NT-enriched slurries.

Table 5 presents the results from the rheology test for the control slurries and the nanomaterial-enriched slurries. As reported by various authors, the use of nanomaterials can change the rheology of cement slurry [8,25] depending on particle size distribution, dosage, colloidal forces, etc. [8]. In most cases, fresh cement slurry will follow the Bingham plastic model and power law model [4]. Therefore, in this study, data fitting was performed against the Bingham plastic model and the power law model, and the coefficient of determination (R^2^) was used to determine the fitting performance [39]. A better fit will have a value of R^2^ close to 1. The results presented in Table 5 show that both models fitted well with the experimental data, with R^2^ values mostly higher than 0.9. The Bingham plastic model showed better performance than the power law model (except at high dosages of GNA, i.e., GNA-5 and GNA-7). Figure 4 presents the fitting results of the Bingham plastic model for various slurries. according to the Bingham plastic model, high dosages of NS and GNA can significantly increase the yield stress of the cement slurry, while according to the power law model, they can significantly increase the consistency index and decrease the power law behavior index. This could be explained by the accelerated production of cement hydration products that increase the structural building ability of the cement slurry. In addition, due to the significantly increased presence of cement hydration products, high dosages of NS and GNA (NS-7, GNA-5, and GNA-7) altered the rheology and flow behavior of the cement slurries more than any other nanomaterial in this study, with relatively low coefficient of determination for both the Bingham model (R^2^_B_) and power law model (R^2^_P_). In contrast, the addition of various dosages of ANA and NT seemed to have a relatively small effect on the rheology of the cement slurries, suggesting that they probably had very little chemical interactions with cement hydration.

**Figure 5:**
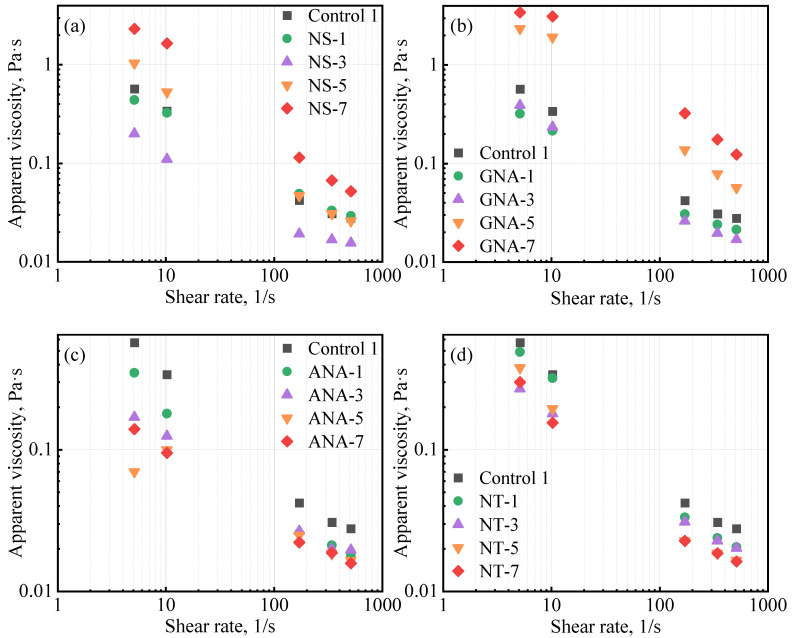
Influence of nanomaterials on class G cement apparent viscosity: (**a**) NS-enriched slurries, (**b**) GNA-enriched slurries, (**c**) ANA-enriched slurries, and (**d**) NT-enriched slurries.

Figure 5 shows the influence of nanomaterials on the apparent viscosity of the cement slurries. The apparent viscosity mostly decreased with increasing shear rate, signifying a shear thinning effect, which is a common fluid behavior for most cement slurries [40]. NS and GNA appeared to have completely opposite effects on cement slurry rheology compared to ANA and NT. For NS and GNA, increasing the dosage from 3% to 7% clearly led to increases in slurry viscosity, while for ANA and NT, increasing the dosage from 1% to 7% clearly led to decreases in slurry viscosity. It should be pointed out that the slurry designs were slightly different, as all ANA- and NT-enriched slurries contained DG as a suspension aid. The higher apparent viscosity of slurry NS-1 and GNA-1 compared to NS-3 and GNA-3, respectively, can be attributed to the presence of DG in the former. Shi et al. also found a decrease in cement slurry viscosity after increasing the dosage of NT from 0% to 1.5% bwoc [41].

**Figure 6:**
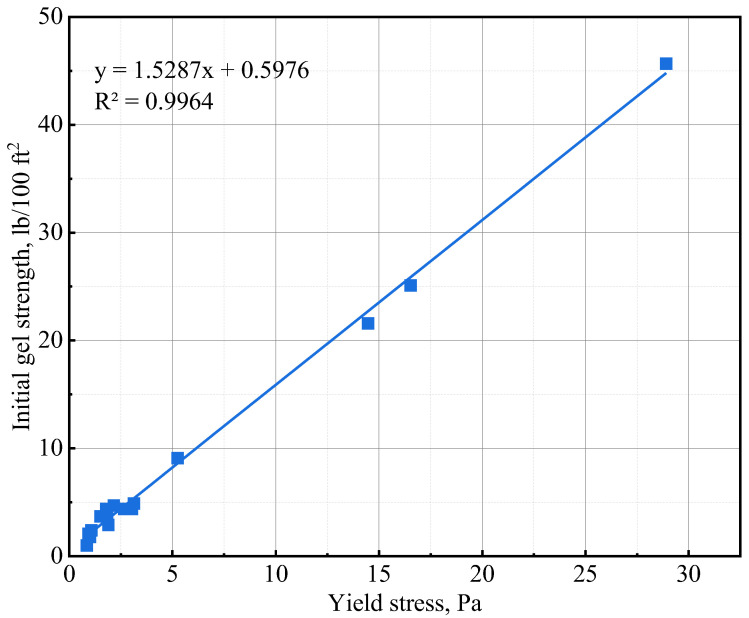
Initial gel strength and yield stress correlation.

The initial gel strength (10 s gel strength) and 10 min gel strength values of the various slurries are presented in Table 6. The addition of NS and GNA to the cement slurries increased the initial and 10 min gel strength most significantly. The increase in gel strength could be primarily attributed to a chemical effect wherein cement hydration is significantly accelerated. The accelerated cement hydration led to the formation of more hydration products and increased the structure building ability of the cement slurry. NS is known to accelerate tricalcium silicate (C_3_S) hydration via a nucleation and pozzolanic effect [42], while GNA is known to accelerate the hydration of tricalcium aluminate (C_3_A) via dissolution to form ettringite [20]. The needle-shaped structure of ettringite probably made GNA a stronger additive in increasing gel strength compared to NS. It is important to note that the lower gel strength of NS-3 compared to that of NS-1 is probably attributable to the presence of suspending agent DG in the NS-1 design. The addition of NT and ANA seemed to have very little influence on the gel strength of the cement slurries, and sometimes, even a slight decrease in gel strength was observed with increasing dosages of NT or ANA. The insignificant influence of NT and ANA on gel strength could be explained by the fact that only a physical effect was associated with the two nanomaterials [28,43], which has little influence on the structural building ability of the cement slurries. As shown in Figure 6, the initial gel strength values obtained in Table 6 exhibit excellent correlation with the yield stress values of the various cement slurries.

### 3.3. Hydration Kinetics Results

#### 3.3.1. Control Slurries

**Figure 7:**
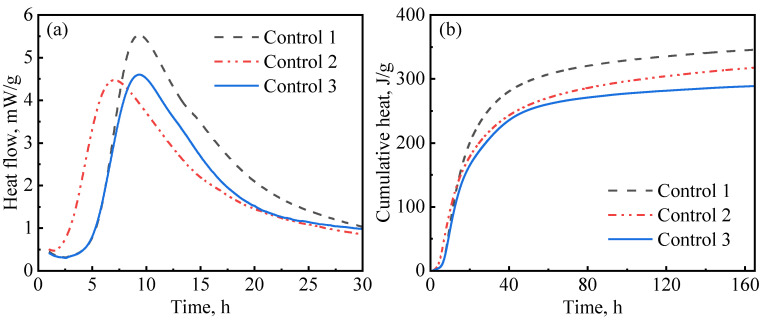
Hydration kinetics of control slurries: (**a**) heat release rate; (**b**) cumulative heat.

In this study, hydration experiments were performed on three control slurry designs (with no nanomaterials). As presented in Table 3, in addition to the defoamer (G603), which was present in all slurries, control-1 contained both a dispersant (PCE) and suspension aid (DG); control-2 only contained a suspension aid (DG), while control-3 only contained a dispersant (PCE). The aim of these designs was to evaluate the influences of the dispersant and suspension aid on hydration kinetics. Figure 7 presents the results for the hydration kinetics of the control slurries. As reported by various authors, PCE has a retardation effect on cement hydration [44]. As can be seen in Figure 7a, control-1 and control-3, which contained PCE, had a long induction period compared to control-2, which had no PCE. These results are similar to what has been previously reported by other authors [45]. Figure 7 also shows that control-1 had the highest hydration peak and the highest cumulative heat of hydration among the three slurries. This phenomenon could be attributed to the fact that the addition of diutan gum in the presence of the superplasticizer resulted in a well-suspended and well-dispersed slurry, thereby enhancing hydration. It has been reported by Pang et al. [15] that suspension aids retain water, increasing the hydration rate at later ages. Similarly, superplasticizers disperse aggregation and release entrapped water, providing the cement particles with even access to water [46,47] and allowing for the even growth of hydration products in the pore spaces [15]. Therefore, after the end of the induction period, the hydration reactions were accelerated, which resulted in a high hydration peak being observed. In contrast, control-3, which also contained PCE but had no suspension aid, showed a lower hydration peak and lower cumulative heat. This could be attributed to the presence of free water resulting from poor slurry suspension, which decreased the amount of mixing water available for hydration. Figure 7a indicates that control-2 had a short induction period. This is because the slurry contained no PCE; hence, there was no retardation to the cement hydration.

#### 3.3.2. Nanomaterial-Enriched Slurries

**Figure 8:**
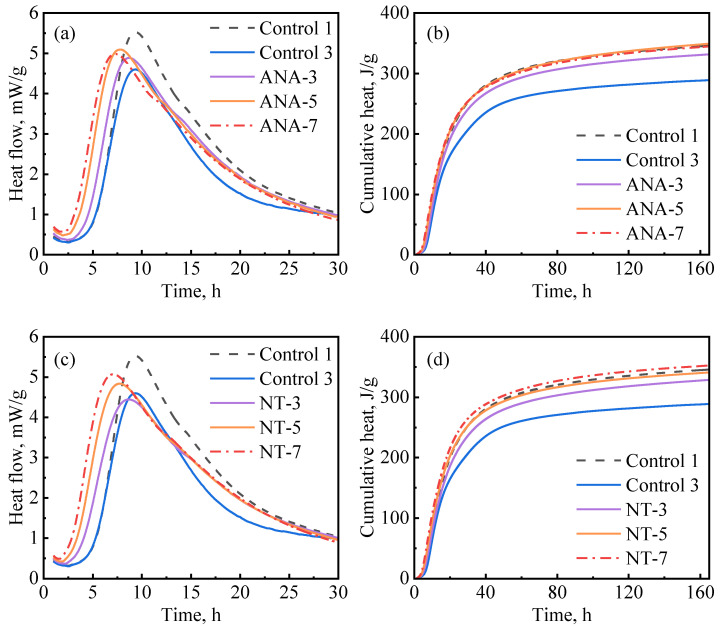
Hydration kinetics of nanomaterial-enriched slurries: (**a**) ANA on heat release rate, (**b**) ANA on cumulative heat flow, (**c**) NT on heat release rate, and (**d**) NT on cumulative heat flow.

Figure 8 depicts the influence of ANA and NT on cement hydration kinetics. As presented in Figure 8a, the hydration kinetics were accelerated with the increase in ANA dosage, as the induction period became shorter and the hydration peak became higher as the dosages increased. NT had a similar effect on hydration kinetics as ANA. The primary difference is that the cumulative hydration heat stopped increasing when the dosage of ANA was increased beyond 5%, while it continued to increase when the dosage of NT was increased from 3% to 7%. Overall, although the ANA- and NT-enriched slurries had significantly increased hydration heat compared with control-3, they exhibited insignificant improvement compared with control-1. Based on the results in Figure 8b–d, 5% bwoc of ANA and 7% bwoc of NT could be considered as optimum dosages in each case.

**Figure 9:**
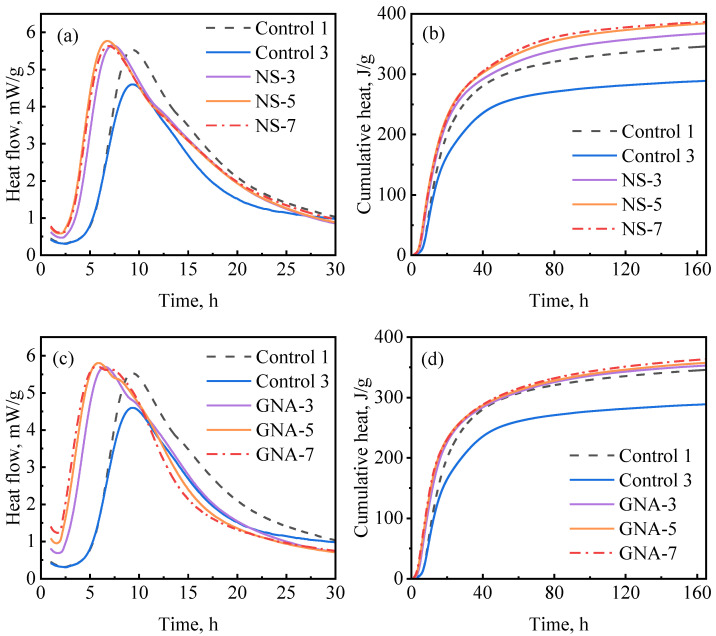
Hydration kinetics of nanomaterial-enriched slurries: (**a**) NS on heat release rate, (**b**) NS on cumulative heat flow, (**c**) GNA on heat release rate, and (**d**) GNA on cumulative heat flow.

Figure 9a,b show the hydration kinetics test results regarding the NS-enriched slurries at different dosages at 30 °C. The addition of NS significantly decreased the induction period, suggesting that NS counteracted the retardation effect of PCE. Figure 9a shows that the induction period and the hydration peak were not significantly influenced by NS dosage in the range from 3% to 7%, while Figure 9b shows that the cumulative hydration heat of the NS-enriched slurries increased with the dosage of NS from 3% to 5% and then remained almost unchanged with further increases in the dosage of NS to 7%. All NS-enriched slurries showed higher cumulative hydration peaks than the well-suspended and well-dispersed optimum control slurry (control-1); this is possibly due to the additional chemical effect (nucleation aid and pozzolanic reaction) exerted by NS. Similar results have also been reported in previous studies [8,48,49].

Figure 9c,d show the hydration kinetics test results regarding the GNA-enriched slurries. The addition of GNA also decreased the induction period (even more significantly than NS). As can be seen in Figure 9c, the induction period and hydration peak were not significantly influenced by the dosage of GNA in the range from 3% to 7%, with only a slight reduction in induction period from 3% to 5% dosage. However, the post-peak behavior was influenced by the dosage of GNA, as the shoulder peak associated with renewed dissolution of the aluminate phase (C_3_A) was clearly accelerated [20]. Similar results were observed by Zhan et al. [50]. Probably because of the relatively low C_3_A content in the cement, the cumulative hydration heat values of the GNA-enriched slurries were only slightly higher than that of the optimum control slurry (control-1) and not significantly influenced by the dosage of GNA in the range from 3% to 7%.

**Figure 10:**
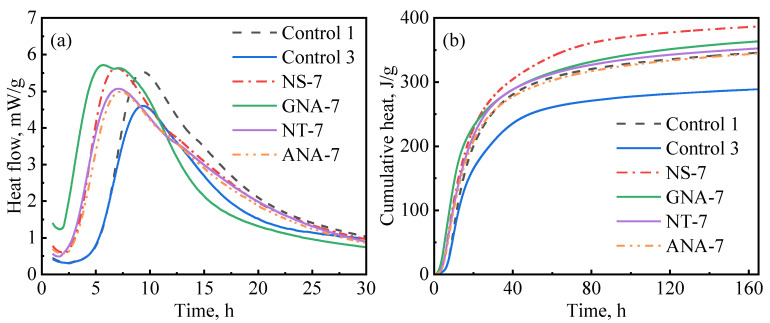
Comparison of nanomaterials on cement hydration kinetics: (**a**) heat release rate; (**b**) cumulative heat.

In general, incorporating nanomaterials into oil well cement accelerates the hydration of cement, but the extent of acceleration depends on different factors, such as the type of nanomaterial, particle size, reactivity, dosage, dispersion, etc. Figure 10 compares the influence of various nanomaterials on cement hydration kinetics at a dosage of 7% bwoc, which represents the maximum acceleration strength (in some cases, there was not much difference between the 5% and 7% dosage). As can be seen in Figure 10a, GNA-7 had the highest hydration peak and shortest induction period compared to the other slurries. Therefore, GNA had the strongest acceleration effect on the early cement hydration kinetics, which can be attributed to two factors: (1) the high dissolution rate of GNA increases alumina ions, promoting ettringite precipitation and C_3_A dissolution; (2) the large surface area of GNA promotes C-S-H nucleation and growth [19,20]. Nevertheless, Figure 10b shows that NS-7 had the highest cumulative heat compared to the other slurries, signifying that NS had the highest overall influence on hydration kinetics in the longer term. C_3_S is known to have the most significant contribution to the hydration heat of cement over the main hydration period, and its hydration rate is primarily controlled by the nucleation and growth of C-S-H. Although all nanomaterials may promote C-S-H nucleation and growth by providing a large surface area, NS seems to have the strongest effect possibly due to its stronger chemical affinity to C-S-H seeds. Despite having the same chemical formula as GNA, the acceleration effect of ANA was much weaker due to its chemical inertness [43]. GNA is generally present in amorphous form with high porosity and high surface area, while ANA is present in stable crystalline form. As can be seen from Table 2, the surface area of GNA is approximately 10 times as high as that of ANA, despite the two having similar particle sizes. As shown in Figure 2, NT and ANA had much higher crystallinity than NS and GNA, which means they have less structural defects and are therefore less reactive. Micro-sized alpha-Al_2_O_3_ was shown to have very limited reaction with Portland cement, even at temperatures as high as 200 °C over long curing periods up to 360 days [51,52]. The high surface area and amorphous structure both contribute to the high chemical reactivity of GNA.

**Figure 11:**
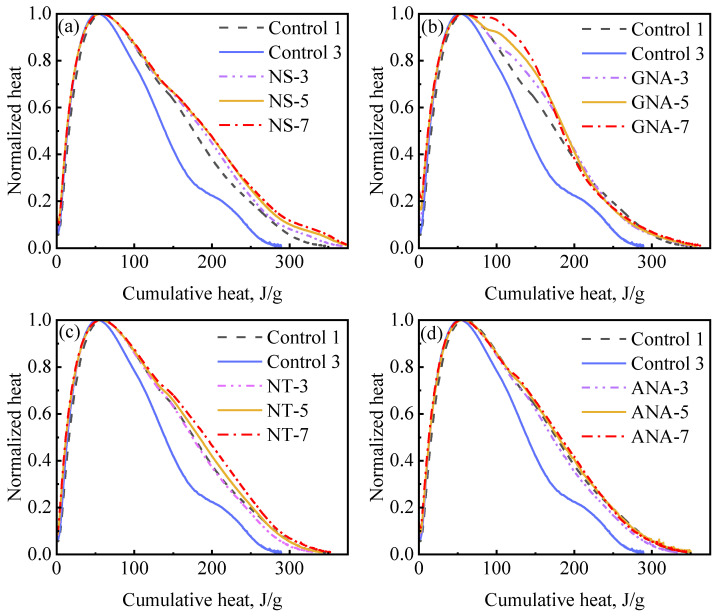
Hydration mechanism profile for different nanomaterial dosages: (**a**) NS-enriched slurries; (**b**) GNA-enriched slurries; (**c**) NT-enriched slurries; (**d**) ANA-enriched slurries.

Cement hydration can be further examined by using hydration mechanism curves, which are also referred to as differential equation curves [53]. Figure 11 presents hydration mechanism curves showing the relation between cumulative hydration heat and normalized heat flow (i.e., heat flow divided by the peak rate) for the nanomaterial-enriched slurries and control slurries. The hydration mechanism curves overlapped during the acceleration period for all slurries, indicating a similar mechanism of reaction. The primary deviations occurred during the deceleration period, where improved slurry suspension significantly increased relative hydration rate at the same hydration extent. These results are consistent with a previous study on the effect of w/c ratio and suspension aids on cement hydration kinetics [53]. The most significant improvement on relative hydration rate was observed due to the addition of DG (from control-3 to control-1). Nanomaterials had a similar but slightly stronger effect than DG in improving the late-stage hydration rate. The accelerated appearance of the shoulder peak due to the incorporation of GNA can also be clearly seen in Figure 11b.

## 4. Prospects of Nanomaterial Applications

Nanomaterials have a wide range of applications in the oil and gas industry due to their unique chemical and physical properties. However, the high cost of nanomaterials limit their large-scale applications. The benefit that nanomaterials bring should always be evaluated when considering the extra cost that their deployment might incur. As shown by the test results of this study, the 7-day hydration extent improvement achieved by the use of nanomaterials was significant compared to a well-dispersed cement slurry system with no suspension aid (control-3). However, the improvement was relatively small compared to a well-dispersed and well-suspended cement slurry system obtained via the combined use of PCE and DG (control-1). Future research should focus on new production methods to reduce the high cost associated with nanomaterials and the potential synergistic effects that can be achieved via the use of nanomaterials and other functional materials.

## 5. Conclusions

For the creation of this paper, the influence of various nanomaterials on the rheological and hydration properties of class G cement in the presence of polycarboxylate superplasticizer was experimentally studied. Based on our study, the following conclusions can be drawn:

The addition of nano-SiO_2_ (NS) and gamma-nano-Al_2_O_3_ (GNA) significantly increased the structure building ability of cement slurries, as evidenced by the increased values of apparent viscosity, gel strength, yield stress (Bingham plastic model), and consistency index (power law model) and a decrease in power law behavior index. This phenomenon can be attributed to the accelerated production of cement hydration products. The addition of NS and GNA altered the rheology and flow behavior of the cement slurries more significantly at higher dosages. In contrast, the addition of alpha-nano-Al_2_O_3_ (ANA) and nano-TiO_2_ (NT) tended to slightly reduce cement slurry rheology.

All nanomaterials can accelerate the early hydration of cement by reducing the induction time, with GNA having the strongest influence. This is attributed to its higher surface area and its ability to accelerate aluminate reactions, which is in great contrast to its chemically inert counterpart, ANA. The dosage of the nanomaterials influences the acceleration strength of ANA and NT more significantly than that of NS and GNA in the range from 3% to 7%.

All nanomaterials can increase the total hydration extent of cement during the late hydration stages due to their ability to mitigate sedimentation and provide better suspension of cement particles, which is similar to the function of a suspension aid (diutan gum). The addition of NS can provide additional increases in hydration extent due to the pozzolanic reaction and its stronger ability to serve as a nucleation aid for C-S-H, while the addition of GNA can accelerate a secondary hydration peak associated with aluminate reactions. The dosage effect on total hydration extent was relatively strong for ANA, NT, and NS in the range from 3% to 5% but weak for GNA from 3% to 7%.

Clearly, ANA and NT influence cement behavior primarily via physical effects, i.e., by increasing the surface area available for the precipitation and growth of C-S-H gel and acting as nano-filling agents. In contrast, NS and GNA can influence cement behavior via both physical and chemical effects, as NS can react with calcium hydroxide to form C-S-H gel (via the pozzolanic reaction) and further promote its nucleation and growth, while GNA can accelerate the aluminate (C_3_A) reaction. For oil well cement with a low C_3_A content, only NS can further increase the 7-day hydration extent compared to a slurry containing both a suspension aid (diutan gum) and polycarboxylate superplasticizer.

## Figures and Tables

**Table 1:** Chemical composition of class G cement in %.

C_3_S	C_2_S	C_3_A	C_4_AF	CaSO_4_	Free Lime
55.73	15.3	2.29	8.61	5.15	0.15

**Table 2:** Nanomaterial properties.

Property	NS	NT	ANA	GNA
Size (nm)	15–25	20–30	0–30	20
Surface area (m^2^/g)	-	30–50	10–20	100–300
Density (g/cm^3^)	2.39	3.9	3.63	3.27

**Table 3:** Slurry composition design.

Slurry	Cement (%)	W/C	NS %bwoc	NT %bwoc	ANA %bwoc	GNA %bwoc	PCE %bwoc	DG %bwoc	G603 %bwoc
C-1	100	0.718	-	-	-	-	1.6	0.02	0.2
C-2	100	0.735	-	-	-	-	-	0.02	0.2
C-3	100	0.718	-	-	-	-	1.6	-	0.2
NS-1	100	0.723	1	-	-	-	1.6	0.01	0.2
NS-3	100	0.73.3	3	-	-	-	1.6	-	0.2
NS-5	100	0.742	5	-	-	-	1.6	-	0.2
NS-7	100	0.752	7	-	-	-	1.6	-	0.2
NT-1	100	0.727	-	1	-	-	1.6	0.01	0.2
NT-3	100	0.745	-	3	-	-	1.6	0.01	0.2
NT-5	100	0.763	-	5	-	-	1.6	0.01	0.2
NT-7	100	0.78	-	7	-	-	1.6	0.01	0.2
ANA-1	100	0.727	-	-	1	-	1.6	0.01	0.2
ANA-3	100	0.743	-	-	3	-	1.6	0.01	0.2
ANA-5	100	0.761	-	-	5	-	1.6	0.01	0.2
ANA-7	100	0.777	-	-	7	-	1.6	0.01	0.2
GNA-1	100	0.726	-	-	-	1	1.6	0.01	0.2
GNA-3	100	0.741	-	-	-	3	1.6	-	0.2
GNA-5	100	0.757	-	-	-	5	1.6	-	0.2
GNA-7	100	0.772	-	-	-	7	1.6	-	0.2

**Table 4:** Sedimentation test results.

	C-3		NS-2		NS-5		NS-7	
Segment	ρ g/cm^3^	Δρ %	ρ g/cm^3^	Δρ %	ρ g/cm^3^	Δρ %	ρ g/cm^3^	Δρ %
1 (Top)	1.23	66.33	1.655	97.01	1.68	99.70	1.680	99.77
2	1.50	81.28	1.706	100.00	1.67	99.11	1.678	99.65
3	1.91	103.45	1.712	100.35	1.68	99.70	1.685	100.07
4	2.07	112.15	1.708	100.12	1.69	100.30	1.686	100.13
5	2.13	115.15	1.719	100.76	1.69	100.30	1.685	100.07
6 (Bottom)	2.28	123.22	1.736	101.76	1.70	100.89	1.689	100.31

**Table 5:** Rheology model fitting results.

	Bingham Plastic Model			Power Law Model		
Slurry	R^2^_B_	μ_p_ (PaS)	τ_y_ (Pa)	R^2^_P_	K (Pas^n^)	n
C-1	0.998	0.0218	3.12	0.969	1.65	0.32
NS-1	0.977	0.0242	3.03	0.991	1.26	0.38
NS-3	1	0.0137	0.99	0.969	0.46	0.43
NS-5	1	0.0157	5.24	0.913	3.67	0.18
NS-7	0.899	0.0244	14.46	0.876	10.52	0.14
NT-1	0.992	0.0170	2.66	0.977	1.35	0.32
NT-3	0.989	0.0174	1.70	0.993	0.68	0.42
NT-5	0.999	0.0130	1.83	0.934	1.05	0.31
NT-7	0.998	0.0136	1.50	0.963	0.75	0.36
ANA-1	0.995	0.0150	1.77	0.962	0.88	0.35
ANA-3	0.994	0.0176	1.06	0.992	0.38	0.50
ANA-5	0.979	0.0156	0.84	0.973	0.17	0.63
ANA-7	0.987	0.0148	0.92	0.995	0.30	0.52
GNA-1	0.995	0.0179	1.89	0.981	0.86	0.39
GNA-3	0.997	0.0130	2.15	0.959	1.20	1.29
GNA-5	0.789	0.0272	16.53	0.845	11.11	0.15
GNA-7	0.774	0.080	28.91	0.917	14.32	0.25

**Table 6:** Gel strength results.

Slurry	Initial Gel Strength lb/100 ft^2^	10 Min Gel Strength lb/100 ft^2^
C-1	4.9	7.9
NS-1	4.4	24.6
NS-3	1.8	12.5
NS-5	9.1	22.9
NS-7	21.6	38.3
NT-1	4.4	15.8
NT-3	3.7	11.7
NT-5	3.7	11.7
NT-7	3.7	11.7
ANA-1	4.4	15.8
ANA-3	2.4	8.5
ANA-5	1	9.5
ANA-7	2.1	9.5
GNA-1	2.9	10.4
GNA-3	4.7	13.3
GNA-5	25.1	31.2
GNA-7	45.7	50.6

## Data Availability

Data will be provided upon request.
